# Uniportal endoscopic posterior lumbar interbody fusion and minimally invasive transforaminal lumbar interbody fusion for elderly patients with lumbar degenerative diseases: a retrospective comparative study of reduced surgical trauma and accelerated early pain relief

**DOI:** 10.3389/fsurg.2026.1784345

**Published:** 2026-03-31

**Authors:** Juanming Lan, Bin Cao, Yongpeng Lin, Weixiong Hu, Rui Lin, Lulu Li, Bolai Chen

**Affiliations:** 1 The Second Clinical College of Guangzhou University of Chinese Medicine, Guangzhou, China; 2 Orthopedics Department, Guangdong Provincial Hospital of Chinese Medicine, Guangzhou, China

**Keywords:** endoscope, lumbar degenerative diseases, lumbar interbody fusion, lumbar vertebrae, minimally invasive

## Abstract

**Background:**

Lumbar degenerative diseases are prevalent spinal disorders among the elderly population worldwide, significantly impairing their quality of life. Both minimally invasive transforaminal lumbar interbody fusion (MIS-TLIF) and uniportal endoscopic posterior lumbar interbody fusion (Endo-PLIF) are established minimally invasive interbody fusion modalities for the management of these conditions. However, MIS-TLIF is associated with limitations such as soft tissue compression induced by dilator tubes and a comparatively prolonged postoperative recovery period. In the present study, we aimed to compare the clinical efficacy and radiographic outcomes of Endo-PLIF vs. MIS-TLIF in the treatment of lumbar degenerative diseases in elderly patients.

**Methods:**

A retrospective analysis was performed of 77 elderly patients with lumbar degenerative diseases who were admitted to the Department of Spinal Minimally Invasive Surgery, Guangdong Provincial Hospital of Traditional Chinese Medicine, between September 2021 and March 2022. The patients were divided into two groups: 35 patients underwent Endo-PLIF and 42 patients underwent MIS-TLIF. The groups were compared in terms of operative time, intraoperative blood loss, length of hospital stay, Visual analogue score (VAS), Oswestry Disability Index (ODI), serum C-reactive protein (CRP) levels, radiographic parameters (including disc space height, lumbar lordosis angle, and fusion rate), the modified MacNab criteria, and the incidence of postoperative complications.

**Results:**

All procedures were successfully completed in both cohorts, with a follow-up period of more than 12 months. No statistically significant difference was observed in operative time between the two groups (*P* *>* *0*.05). The Endo-PLIF group demonstrated significantly superior outcomes regarding intraoperative blood loss, postoperative drainage volume and length of hospital stay (*P* < 0.05). Significant improvements in VAS scores and ODI were observed at all postoperative time points compared with preoperative baseline values in both groups (*P* < 0.05). The Endo-PLIF group exhibited significantly lower low back pain VAS scores at 1 week, 1 month, and 3 months postoperatively, as well as a superior ODI at 3 months, compared with the MIS-TLIF group (*P* *<* 0.05). However, no significant intergroup differences were detected in low back pain VAS scores, ODI at other time points, or leg pain VAS scores across all assessed time points (*P* > 0.05). Furthermore, serum CRP levels at all postoperative intervals were significantly lower in the Endo-PLIF group than in the MIS-TLIF group (*P* < 0.05). No statistically significant differences were noted between the groups in terms of complication rates, the excellent-good rate based on the modified MacNab criteria at final follow-up, postoperative disc space height, postoperative lumbar lordosis angle, or the 12-month fusion rate (94% in the Endo-PLIF group and 95% in the MIS-TLIF group, *P* > 0.05).

**Conclusion:**

Both Endo-PLIF and MIS-TLIF achieve favorable short- to mid-term clinical and radiographic outcomes in elderly patients with lumbar degenerative diseases. Moreover, the Endo-PLIF technique offers additional advantages, including minimal invasiveness, reduced postoperative drainage, and facilitation of accelerated postoperative recovery.

## 1 Background

Lumbar degenerative disease (LDD) is a primary cause of chronic low back pain and neurological dysfunction among the global elderly population (1). It primarily encompasses lumbar disc herniation, lumbar spinal stenosis, lumbar spondylolisthesis, and lumbar instability (2). With the global aging process accelerating, the prevalence of LDD continues to rise, significantly impairing the quality of life of older adults (3). Surgical intervention is recommended when conservative treatment fails or when severe neurological deficits are present (4). Compared with younger patients, elderly individuals have a higher incidence of postoperative complications and require a longer recovery period (5). Therefore, the selection of a surgical approach that minimizes surgical trauma and maximizes safety is crucial for improving clinical outcomes in elderly patients. Lumbar interbody fusion is an effective surgical procedure for the management of LDD that effectively achieves neural decompression, restores intervertebral height, and improves lumbar function (6). Although traditional posterior lumbar interbody fusion (PLIF) and transforaminal lumbar interbody fusion (TLIF) can provide adequate decompression, they are plagued by limitations including extensive soft tissue dissection, prolonged postoperative recovery, and a relatively high complication rate (7, 8).

To minimize surgical trauma, minimally invasive techniques have garnered increasing attention in spinal surgery. In 2002, Foley and Lefkowitz introduced minimally invasive transforaminal lumbar interbody fusion (MIS-TLIF). This procedure allows decompression and fusion to be performed through a small incision via a working sleeve, thereby minimizing injury to the paraspinal muscles and bony structures compared with traditional open surgery. It confers advantages such as minimal invasiveness, reduced intraoperative blood loss, and accelerated postoperative recovery, while achieving clinical outcomes comparable to those of conventional open techniques (9, 10). However, MIS-TLIF is also accompanied by inherent limitations, including a confined working space and a restricted surgical field of view. Furthermore, the use of sequential dilators may lead to compressive injury of the surrounding muscles and result in a relatively prolonged postoperative recovery period (11). In recent years, with advancements in spinal endoscopic technology and refinement of endoscopic instruments, endoscopic posterior lumbar interbody fusion (Endo-PLIF) has emerged as a novel therapeutic modality for lumbar degenerative diseases. It enables direct decompression of bony spinal stenosis and safe interbody fusion under endoscopic visualization (12). Guided by the Kambin's triangle principle, our team utilizes an uniportal endoscopic system through a posterolateral approach. Under full endoscopic guidance, facetectomy, neural decompression, and interbody fusion are accomplished. Preliminary findings have demonstrated that Endo-PLIF is a safe, minimally invasive procedure for the management of degenerative lumbar diseases, with excellent short-term efficacy and a low complication rate (13).

However, there is a scarcity of evidence-based comparative studies focused on elderly patients with lumbar degenerative diseases. To address this knowledge gap, this retrospective comparative study was conducted to systematically evaluate and compare the clinical efficacy, radiographic outcomes, and safety profiles of Endo-PLIF and MIS-TLIF in the treatment of this specific patient population. The results aim to offer an objective reference for the selection of minimally invasive fusion strategies.

## 2 Clinical data

### 2.1 Case inclusion and exclusion criteria

The inclusion criteria were as follows: (1) Age ≥ 65 years, with a confirmed diagnosis of lumbar disc herniation with instability, lumbar spinal stenosis with instability, or grade I lumbar spondylolisthesis; (2) Clinical manifestations consistent with radiographic findings, with failure to achieve symptom improvement or even symptom exacerbation after at least 3 months of standardized conservative treatment; (3) Undergoing single-segment decompression and internal fixation surgery using either Endo-PLIF or MIS-TLIF, with complete preoperative and postoperative clinical and radiographic data; (4) All surgeries performed by the same lead surgeon.

The exclusion criteria were as follows: (1) Concurrent spinal disorders including spinal tumors, infection, tuberculosis, spinal deformity, severe osteoporosis, or ankylosing spondylitis; (2) Prior lumbar spine surgical history; (3) Incomplete clinical data or a follow-up period of less than 12 months.

### 2.2 General information of patients in the two groups

This was a retrospective observational study. A total of 77 patients who underwent surgical treatment for lumbar degenerative diseases at our institution between September 2021 and March 2022 were enrolled. Among them, 42 patients underwent MIS-TLIF (MIS-TLIF group) and 35 patients underwent Endo-PLIF (Endo-PLIF group). The two groups showed no statistically significant differences in age, gender, body mass index (BMI), affected spinal segment, disease duration, or disease type (*P* > 0.05, Table 1). The Endo-PLIF group consisted of 7 patients with lumbar disc herniation with instability, 12 patients with lumbar spondylolisthesis, and 16 patients with lumbar spinal stenosis with instability. The MIS-TLIF group consisted of 13 patients with lumbar disc herniation with instability, 14 patients with lumbar spondylolisthesis, and 15 patients with lumbar spinal stenosis with instability.

**Table 1:** Patient basic information.

Characteristics	Endo-PLIF group	MIS-TLIF group	*P*
Gender (male/female)	9/26	12/30	0.779
BMI(kg/m^2^)	24.50 (22.60, 26.70)	24.50 (22.30, 26.05)	0.866
Age (years)	71.3 ± 3.0	72.8 ± 4.1	0.07
Levels			0.402
L3/4	1	2	
L4/5	27	36	
L5/S1	7	4	
Disease duration (months)	12.00 (4.00, 84.00)	12.00 (6.00, 78.00)	0.711
Clinical diagnosis			0.506
Lumbar disc herniation	7	13	
Lumbar spondylolis thesis	12	14	
Lumbar spinal stenosis	16	15	

### 2.3 Surgical methods

To prophylaxis against surgical site infection, 2 g of cefazolin sodium was routinely administered intravenously 30 min preoperatively. An additional 2 g dose was administered intraoperatively if the surgical procedure exceeded 3 h. All patients underwent general anesthesia via endotracheal intubation and were placed in the prone position. Soft cushions were used to support and elevate the chest and bilateral anterior superior iliac spines. Preoperative lateral x-ray fluoroscopy was performed to verify the target surgical segment.

#### 2.3.1 MIS-TLIF group (Using the L4/5 level as an example)

A 3-cm longitudinal incision was made just 2 cm lateral to the posterior midline. After sequentially cutting through the skin, subcutaneous tissue, and deep fascia, the paraspinal muscles were bluntly dissected along the interspinous cleft. The operative channel was sequentially dilated with tubular dilators and ultimately maintained with a Quadrant retractor system to adequately expose the facet joints. The entry sites for the superior and inferior pedicle screws were localized. A pilot hole was drilled through the pedicle into the vertebral body with a hand awl, followed by the insertion of a guidewire. After satisfactory guidewire positioning was confirmed by fluoroscopy, the pedicle screw entry sites were occluded with bone wax. Through the working channel, facetectomy, ligamentum flavum excision, and nerve root decompression were accomplished with osteotomes and laminectomy rongeurs. The intervertebral disc space was prepared, and the interbody cage pre-loaded with autologous bone graft was implanted. Polyaxial pedicle screws were subsequently inserted. An appropriately sized titanium rod was cut, contoured, placed, and tightened to the screws with compression applied. Final fluoroscopic examination verified the satisfactory placement of all implants. The locking screws were tightened, a drainage tube was inserted at the decompression site, and the wound was closed in layers.

#### 2.3.2 Endo-PLIF group (Using the L4/5 level as an example)

Two 2-cm transverse skin incisions were made laterally at the projected entry sites of the bilateral pedicles. Pedicle screw guidewires were inserted, and percutaneous pedicle screws were first placed on the non-decompression side. On the symptomatic side, an endoscopic reamer sleeve was inserted and secured in place, with its positioning over the L4 inferior articular process verified by fluoroscopy. Under endoscopic guidance, soft tissues were dissected to expose the medial border and tip of the inferior articular process. The reamer was utilized to partially resect the L4 inferior articular process and lamina in a stepwise fashion. After hemostasis was achieved with a radiofrequency probe, the ligamentum flavum and the tip of the L5 superior articular process were exposed. A portion of the L5 superior articular process tip was excised with rongeurs, and the harvested autologous bone fragments were retained.

Along the superior margin of the L5 pedicle, the nerve root was gently retracted and safeguarded outside the working channel using the cannula. The annulus fibrosus was incised with an osteotome. The intervertebral disc space was subsequently prepared with a combination of pituitary rongeurs, detachable reverse-mounted reamers, push-pull chisels, and crescent-shaped reamers. The cartilage endplates were meticulously curetted until punctate bleeding from the bony surface was observed, confirming adequate endplate preparation. With an osteotome, partial vertebral bone fragments were chiseled from the anterior vertebral body margin to form an overlapping “fish-scale” pattern, ensuring contact between these bone fragments at the anterior aspect of the intervertebral disc space (the “Fish Scale” technique). The endoscope was subsequently withdrawn. A mixture of autologous and allogeneic bone graft was implanted and tightly impacted into the intervertebral disc space using a bone graft funnel and tampers (the “Rammed Earth” technique).

**Figure 1:**
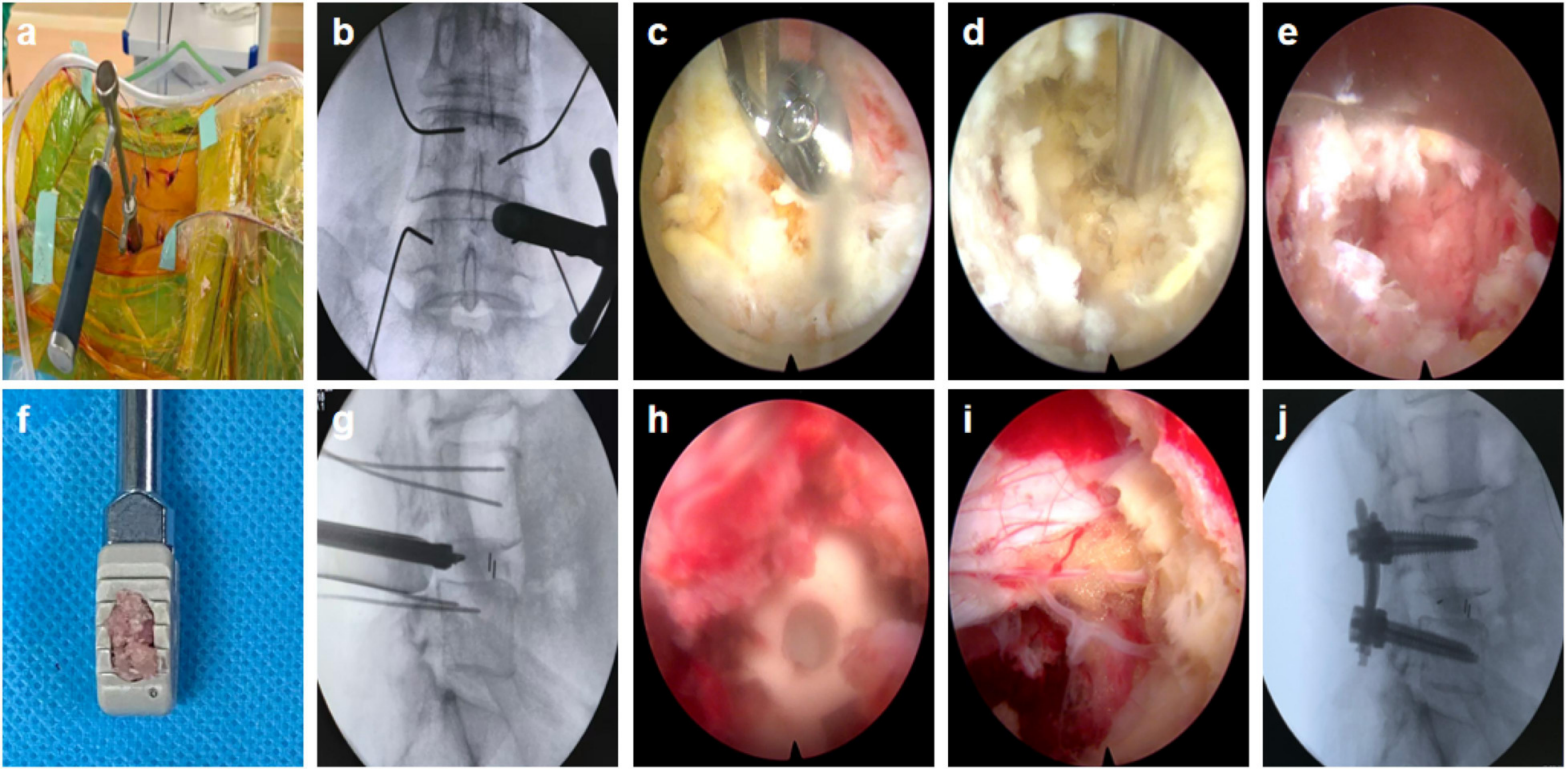
Surgical procedure of Endo-PLIF establishment of the working channel **(a,b)**. Endoscopic ipsilateral intraspinal decompression **(c)**. Disc space preparation and graft bed preparation **(d,e)**. Interbody grafting and cage implantation **(f,g,h)**. Ligamentum flavum resection **(i)**. Percutaneous pedicle screw insertion **(j)**.

The assembled working sheath was reinserted. Under endoscopic guidance and following confirmation of the absence of neurovascular structures, the thecal sac and nerve root were gently retracted to facilitate interbody cage implantation, with its final positioning verified by fluoroscopy. Hemostasis was accomplished under endoscopic guidance, and the nerve root was inspected. Central canal decompression was completed by resecting the residual ligamentum flavum. The working channel was subsequently removed. On the decompression side, pedicle screws were inserted percutaneously along the guidewires. The pre-contoured titanium rod was placed, compressive force was applied across the segment, and the screws were ultimately locked. A drainage tube was placed at the decompression site, and the wound was closed in layers. The surgical workflow is illustrated in Figure 1.

### 2.4 Postoperative management

The postoperative management protocol was identical in both groups. All patients were administered 2 g of cefazolin sodium intravenously for infection prophylaxis, along with routine adjunctive medications including analgesics, anti-inflammatory agents, neurotrophic drugs, and gastrointestinal protectants. Patients were advised to conduct straight leg raising exercises on the first postoperative day. The draining tube was removed when the drainage volume was < 50 ml/24 h. On the second postoperative day, patients were encouraged to ambulate while wearing a lumbosacral orthosis. The orthosis was worn for a maximum of 3 months postoperatively.

### 2.5 Outcomes

The operative time, postoperative drainage volume, intraoperative blood loss, length of hospital stay, and incidence of complications (including iatrogenic dural tears, surgical site infection, dysesthesia, and nerve root injury) were documented for both groups. Estimation of intraoperative blood loss: Intraoperative blood loss was not accurately quantifiable in the Endo-PLIF group, as the entire surgical procedure was performed in a fluid irrigation medium. Thus, the volume of intraoperative blood loss was indirectly estimated by subtracting the total volume of normal saline used for intraoperative irrigation from the total volume of aspirated fluid collected from the surgical field intraoperatively. By contrast, intraoperative blood loss in the MIS-TLIF group was assessed using conventional clinical methods. Serum C-reactive protein (CRP) levels were measured preoperatively and on postoperative days 1, 3, and 5 to assess the degree of surgical trauma and associated stress response. Clinical outcomes were evaluated with the Visual Analogue Scale (VAS) for low back pain and leg pain, the Oswestry Disability Index (ODI), and the modified MacNab criteria. The lumbar lordotic angle (LL) and intervertebral disc height (DH) were measured preoperatively as well as at 3 and 12 months postoperatively. At the 12-month follow-up, interbody fusion was assessed based on the Bridwell grading system: Grade I, complete fusion with trabecular bone bridging; Grade II, graft consolidation without complete trabecular bridging but no radiolucent line; Grade III, graft consolidation with a distinct radiolucent line; Grade IV, definite nonunion with graft resorption and intervertebral space collapse. All radiographic measurements were independently performed by two attending spine surgeons on three separate occasions, and the mean values were included for statistical analysis.

### 2.6 Statistical analysis

Statistical analyses were performed using SPSS software (version 22.0). Continuous data that conformed to a normal distribution were presented as mean ± standard deviation (SD), and intergroup comparisons were conducted using the independent samples t-test. Continuous data that did not follow a normal distribution were presented as median and interquartile range (IQR), with intergroup comparisons performed using the rank-sum test. For intragroup comparisons across different time points, repeated-measures analysis of variance (ANOVA) was used for normally distributed data, while the Friedman test was applied for non-normally distributed data. Categorical data were presented as frequencies and percentages (%), and intergroup comparisons were performed using the *χ*² test or Fisher's exact test, as appropriate. *P* *<* 0.05 was considered statistically significant.

## 3 Results

All surgical procedures were successfully performed in both groups. Data on operative time, intraoperative blood loss, length of hospital stay, and postoperative drainage volume for both groups are presented in Table 2. No statistically significant difference was noted in operative time between the two groups (*P* > 0.05). The Endo-PLIF group exhibited significantly less intraoperative blood loss, lower postoperative drainage volume, and a shorter hospital stay compared with the MIS-TLIF group.(*P* < 0.05).

**Table 2:** Perioperative parameters.

Perioperative parameters	Endo-PLIF group	MIS-TLIF group	*P*
Operative time (minutes)	194.4 ± 43.0	175.8 ± 45.0	0.07
Intraoperative blood loss (mL)	50.0 (30.0, 100.0)	162.50 (100.00, 200.0)	<0.001
Drainage volume(mL)	127.42 ± 32.34	188.40 ± 39.26	<0.001
Hospital stays(day)	9.00 (7.00, 11.00)	10.00 (8.00, 13.00)	0.04

**Figure 2:**
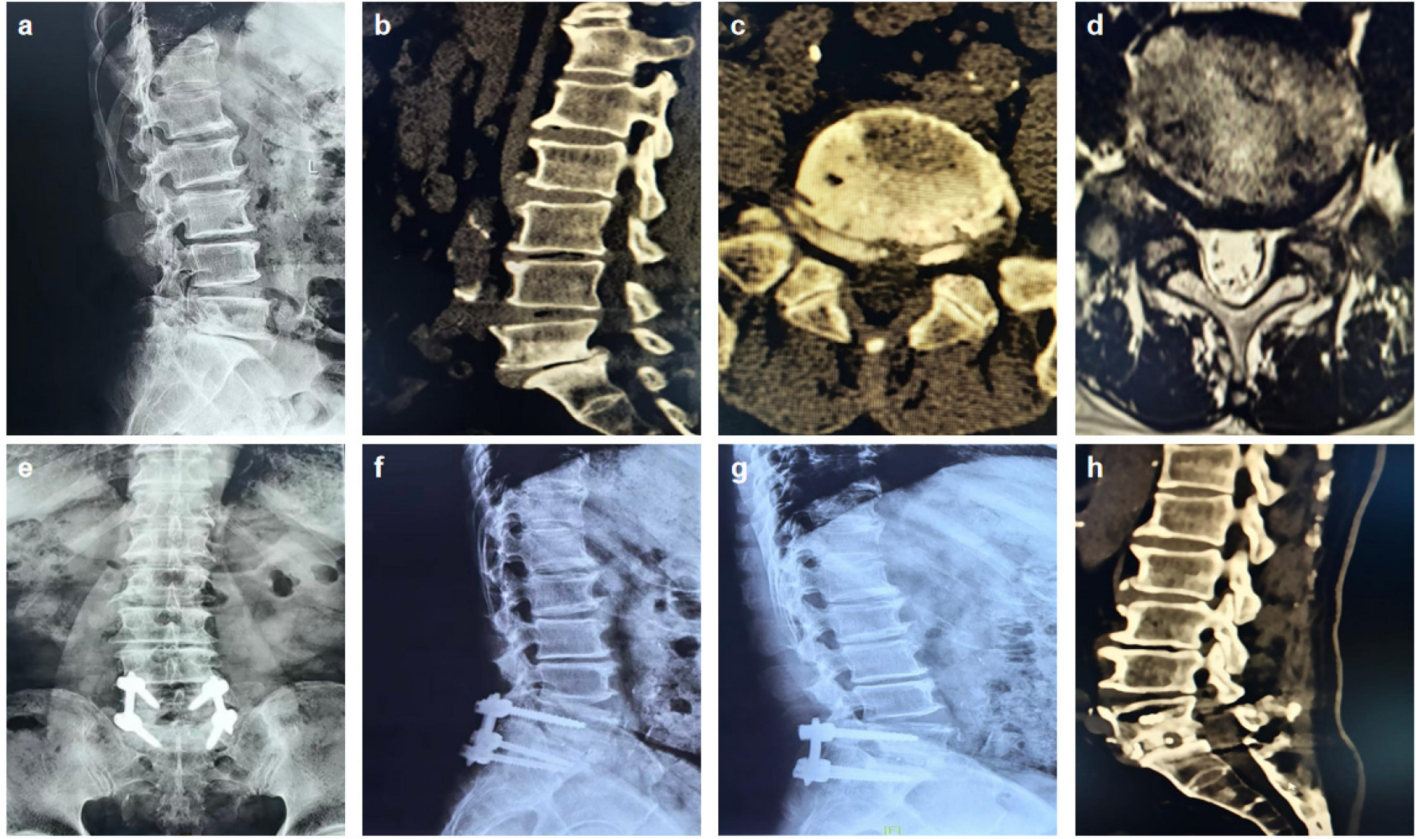
Imaging data of a typical case in Endo-PLIF A 72-year-old male patient. Preoperative DR, CT and MRI show grade I lumbar spondylolisthesis **(a,b,c,d)**. Postoperative 3-month X-rays show well-positioned pedicle screws and interbody cage **(e,f)**. Postoperative 12-month X-ray demonstrates maintained satisfactory positional stability of the cage and implants **(g)**. Postoperative 12-month CT scans confirm satisfactory positioning of the implants and solid bony interbody fusion at Bridwell Grade I **(h)**.

All 77 patients were followed up for more than 12 months (Table 3). Both groups achieved significant improvements in low back pain Visual Analogue Scale (VAS) scores, leg pain VAS scores, and Oswestry Disability Index (ODI) scores when compared with their preoperative counterparts (*P* < 0.05). At 1 week, 1 month, and 3 months postoperatively, the Endo-PLIF group had significantly lower low back pain VAS scores than the MIS-TLIF group (*P* < 0.05). However, no statistically significant intergroup differences were noted at 6 and 12 months postoperatively (*P* > 0.05). Similarly, there were no statistically significant differences in leg pain VAS scores between the two groups at any postoperative time points (1 week, 1 month, 3 months, 6 months, and 12 months; *P* > 0.05). At the 3-month follow-up, the Endo-PLIF group had significantly lower ODI scores than the MIS-TLIF group (*P* < 0.05), with no statistically significant differences observed at 6 and 12 months postoperatively (*P* > 0.05). Serum C-reactive protein (CRP) levels were significantly elevated postoperatively in both groups relative to preoperative levels (*P* < 0.05). Notably, the Endo-PLIF group had significantly lower CRP levels at all postoperative time points than the MIS-TLIF group (*P* < 0.05). At the final follow-up, based on the modified MacNab criteria, the Endo-PLIF group had 18 excellent, 15 good, and 2 fair outcomes, yielding an excellent-to-good rate of 94.3%. The MIS-TLIF group had 21 excellent, 17 good, and 4 fair outcomes, with an excellent-to-good rate of 90.5%. The difference in the excellent-to-good rate between the two groups was not statistically significant (*P* > 0.05). Representative radiographic images of a typical case in the Endo-PLIF group are presented in Figure 2.

**Table 3:** Comparison of follow-up outcomes in group Endo-LIF and group MIS-TLIF.

Comparison of follow-up outcomes	Endo-PLIF group	MIS-TLIF group	*P*
VAS of low-back pain			
Preoperative	5.00 (4.00, 6.00)	5.00 (4.00, 6.00)	0.692
1 week postoperative	3.00 (2.00, 3.00)*	3.00 (2.00, 5.00)*	0.008
1 month postoperative	2.00 (2.00, 3.00)*	3.00 (2.00, 3.00)*	0.024
3 months postoperative	2.00 (2.00, 3.00)*	2.50 (2.00, 3.00)*	0.007
6 months postoperative	2.00 (2.00, 2.00)*	2.00 (2.00, 2.00)*	0.68
12 months postoperative	1.00 (1.00, 1.00)*	1.00 (1.00, 2.00)*	0.87
VAS of leg pain			
Preoperative	6.00 (5.00, 7.00)	6.00 (5.00, 7.00)	0.707
1 week postoperative	2.00 (2.00, 3.00)*	2.50 (2.00, 3.00)*	0.194
1 month postoperative	2.00 (1.00, 2.00)*	2.00 (1.00, 2.00)*	0.812
3 months postoperative	1.00 (1.00, 2.00)*	2.00 (1.00, 2.00)*	0.295
6 months postoperative	1.00 (0.00, 1.00)*	1.00 (0.75, 1.00)*	0.441
12 months postoperative	0.00 (0.00, 1.00)*	0.00 (0.00, 1.00)*	0.346
ODI			
Preoperative	50.00 (44.00, 60.00)	48.00 (44.50, 58.50)	0.951
3 months postoperative	18.00 (16.00, 24.00)*	24.00 (18.00, 32.00)*	0.02
6 months postoperative	16.00 (12.00, 18.00)*	15.00 (12.00, 20.00)*	0.844
12 months postoperative	10.00 (8.00, 14.00)*	12.00 (10.00, 15.25)*	0.346
CRP(mg/L)			
Preoperative	1.21 (0.86, 1.78)	1.26 (0.40, 2.04)	0.83
1 day postoperative	16.24 (11.28, 24.34)*	20.96 (15.42, 25.62)*	0.044
3 days postoperative	58.62 (52.70, 67.52)*	77.16 (64.85, 97.34)*	<0.001
5 days postoperative	36.52 (27.85, 44.52)*	53.3 (42.65, 63.10)*	<0.001
MacNab	18:15:2:0	21:17:4:0	>0.05

Abbreviations: VAS, visual analogue scale; ODI, oswestry disability index; CRP, C-reactive protein.

* Compared with preoperative, *P* < 0.05.

Both groups achieved significant improvements in lumbar lordotic angle (LL) and intervertebral disc height (DH) when compared with their preoperative counterparts (*P* < 0.05). No statistically significant intergroup differences in these radiographic parameters were noted at any postoperative time points (*P* > 0.05). According to the Bridwell interbody fusion grading criteria, the Endo-PLIF group comprised 33 cases classified as fused and 2 cases classified as probably fused, yielding a fusion rate of 94.3%. The MIS-TLIF group comprised 40 cases classified as fused and 2 cases classified as probably fused, yielding a fusion rate of 95.2%. The difference in fusion rates between the two groups was not statistically significant (*P* > 0.05). Detailed data are presented in Table 4.

**Table 4:** Comparison of preoperative and follow-up imaging data between the two groups.

Comparison of imaging data	Endo-PLIF group	MIS-TLIF group	*P*
LL			
Preoperative	33.94 ± 9.94	34.10 ± 11.10	0.947
3 months postoperative	39.89 ± 7.05*	40.70 ± 9.62*	0.672
12 months postoperative	40.30 ± 5.55*	41.23 ± 6.27*	0.496
DH			
Preoperative	9.05 ± 1.65	9.29 ± 2.64	0.628
3 months postoperative	11.21 ± 1.38*	11.43 ± 2.08*	0.591
12 months postoperative	10.87 ± 1.30*	10.82 ± 1.88*	0.868
Interbody fusion rate	33 (94.3%)	40 (95.2%)	>0.05

Abbreviations: LL, lumbar lordosis; DH, disc height.

* Compared with preoperative, *P* < 0.05.

There was no statistically significant difference in the complication rate between the two groups.In the Endo-PLIF group, two postoperative complications occurred (complication rate: 5.7%). One case involved exacerbation of lower limb numbness, which was attributed to intraoperative nerve root compression. Symptoms were alleviated following symptomatic treatment including neurotrophic therapy, analgesics, and anti-inflammatory agents, and the numbness had completely resolved by the 3-month follow-up. The other case was a dural tear, which healed with conservative management. In the MIS-TLIF group, three postoperative complications occurred (complication rate: 7.1%). Two cases were surgical site infections, which resolved after an extended course of antibiotics and enhanced wound management. One case was a dural tear, which also healed with conservative management.

## 4 Discussion

Elderly patients often present with multiple comorbidities and exhibit impaired tolerance to surgical trauma (14). Therefore, minimizing surgical trauma, accelerating postoperative recovery, and ensuring therapeutic efficacy constitute the core objectives of surgical management for this specific population. In recent years, with advancements in minimally invasive lumbar spine techniques, the core technical workflow of Endo-PLIF has become increasingly standardized, surgical efficiency has steadily improved, and the scope of applicable pathologies has been expanded (15). This study compared Endo-PLIF with MIS-TLIF to investigate their clinical utility in the treatment of single-segment lumbar degenerative diseases in elderly patients. Our results demonstrate that compared with the MIS-TLIF group, the Endo-PLIF group was associated with less intraoperative blood loss, lower postoperative drainage volume, a shorter length of hospital stay, and more pronounced improvements in low back pain during the early postoperative period.

The Endo-PLIF group had significantly less intraoperative blood loss than the MIS-TLIF group (*P* < 0.05).This finding can be attributed to the through-translation technique employed in the single-channel endoscopic approach, which minimizes soft tissue trauma from dissection. Furthermore, the entire procedure was conducted under direct endoscopic visualization in a fluid medium. The magnified endoscopic visualization, combined with hydrostatic pressure effects, helped reduce surgical site bleeding, thereby leading to a lower postoperative drainage volume.

The shorter length of hospital stay in the Endo-PLIF group was primarily attributed to more pronounced postoperative pain relief and earlier drainage tube removal. Our results showed that the Endo-PLIF group had more favorable low back pain VAS scores in the early postoperative period and a lower ODI at 3 months compared with the MIS-TLIF group (*P* < 0.05), indicating that Endo-PLIF confers advantages in early pain relief and functional recovery. Yang et al. (16) in their comparison of the short-term efficacy of Endo-PLIF vs. traditional PLIF for lumbar spinal stenosis in elderly patients, demonstrated that both groups achieved significant reductions in postoperative VAS and ODI scores, with Endo-PLIF exhibiting superior early outcomes. A retrospective analysis by Chen et al. (17) of 99 patients with lumbar degenerative diseases (LDD) also reported lower low back pain VAS scores at all time points in the Endo-LIF group compared with the MIS-TLIF group, which aligns with our results. The need for paraspinal muscle dissection and compressive damage to surrounding soft tissues caused by sequential dilators in MIS-TLIF may induce a more prominent surgical trauma-related stress response. In contrast, Endo-PLIF requires only four small incisions, rendering a more minimally invasive strategy with less soft tissue injury. Furthermore, by employing the Kambin's triangle approach under endoscopic guidance, Endo-PLIF enables targeted removal of pathological bony structures and the ligamentum flavum without extensive paraspinal muscle dissection. This significantly minimizes denervation atrophy and scar adhesions caused by muscle dissection, effectively alleviating early postoperative pain and reducing complications such as lumbar stiffness, thereby promoting patient recovery.

C-reactive protein (CRP) is a sensitive and objective clinical indicator for evaluating surgical trauma-associated stress, with its level directly reflecting the extent of tissue damage and the intensity of the inflammatory response (18). In our study, CRP levels at all postoperative time points were significantly lower in the Endo-PLIF group than in the MIS-TLIF group (*P* < 0.05), suggesting that Endo-PLIF results in less surgical trauma. This constitutes one of the primary reasons why Endo-PLIF can facilitate postoperative recovery. This finding is also consistent with the findings of a prospective study by AO et al. (19).

Regarding operative time, no statistically significant difference was noted between the two groups (*P* > 0.05), despite the relatively longer operative time observed in the Endo-PLIF group. This finding aligns with the results reported by Yuan et al. (20) and may be attributable to the relatively steep learning curve of Endo-PLIF. This technique demands a high degree of precision in endoscopic manipulation, as tasks including nucleus pulposus resection, endplate preparation, and interbody grafting need to be accomplished within the confined working channel. Limited instrument maneuverability and procedural complexity are responsible for the prolonged operative time. It is expected that with improved surgical proficiency and continuous refinement of endoscopic instruments, the efficiency of this technique will be enhanced.

Traditional lumbar fusion surgery is considered the gold standard for lumbar fusion, as it can restore spinal physiological alignment, achieve thorough decompression, and yield satisfactory fusion rates (21). However, the long-term fusion quality of endoscopic lumbar fusion remains a matter of debate, with some scholars questioning whether its interbody fusion rate is comparable to that of traditional open fusion. A retrospective study by He et al. (22) in patients with single-segment lumbar degenerative diseases (LDD) demonstrated comparable fusion rates between the endoscopic fusion group (93.3%) and the traditional open group (96.7%). Both groups also successfully restored lumbar lordotic angle (LL) and intervertebral disc height (DH). This finding was corroborated by a retrospective study by Zhang et al. (23), which reported no statistically significant difference in fusion rates between the endoscopic lumbar fusion group (93.8%) and the MIS-TLIF group (100%).

Our results are consistent with the abovementioned studies. Both groups showed significant improvements in LL and DH postoperatively when compared with their preoperative counterparts (*P* < 0.05), with no statistically significant difference between the two groups (*P* > 0.05). This indicates that the Endo-PLIF group achieved comparable efficacy to the MIS-TLIF group in restoring spinal alignment and intervertebral disc height. The fusion rates at 12 months postoperatively did not differ significantly (*P* > 0.05), and no cases of cage migration or subsidence were observed in either group.The high fusion rate achieved in the Endo-PLIF group in this study is attributed to the innovative techniques employed by our team, namely the “Rammed Earth” and “Fish Scale” techniques, which enable more effective interbody bone grafting and enhance bony fusion.Consensus in the field holds that optimal endplate preparation entails thorough debridement of endplate cartilage with preserved subchondral bone integrity and evident punctate bleeding on the subchondral surface (24). Both endplate preparation quality and bone grafting area are key determinants critically governing interbody fusion outcomes (25). Guided by the core principle that microbleeding from adequate endplate preparation augments interbody fusion, our team developed the novel “Fish Scale” endplate preparation technique. Specifically, artificial microfractures are created on the subchondral bone of the superior and inferior endplates using a small osteotome at the non-weight-bearing region of the anterior vertebral endplate margin; the chiseled bone fragments—each less than 2 mm in thickness—assume a fish-scale appearance, with one end anchored to the endplate and the free end everted anteriorly toward the anterior intervertebral disc space annulus fibrosus. Our clinical experience demonstrates that multi-angle, extensive discectomy combined with meticulous endplate preparation achieves more thorough intervertebral disc space preparation, thus maximizing the bone grafting area. Concurrently, microfractures and subsequent microbleeding induced by the “Fish Scale” technique effectively accelerate bone graft fusion and facilitate the formation of a stable anterior column bony bridge at the anterior intervertebral disc space margin.

Furthermore, our team adopted the complementary “Rammed Earth” bone grafting technique, whereby a mixture of autologous and allogeneic bone graft is tightly compacted into the prepared intervertebral disc space using a bone graft funnel and tamper. This maneuver eliminates intra-discal dead space, bolsters the initial stability of the bone graft and fusion cage, and further elevates the bone graft fusion rate by increasing contact pressure between the graft material and prepared endplate surface.

A meta-analysis (26) reported that the complication rate of endoscopic lumbar fusion was 5.9%, which is comparable to that of MIS-TLIF (7.0%). In our study, the complication rate was 5.7% (2 cases) in the Endo-PLIF group and 7.1% (3 cases) in the MIS-TLIF group, with no statistically significant difference noted between the two groups (*P* > 0.05). Importantly, no severe complications such as nerve root injury occurred, confirming that Endo-PLIF is a relatively safe minimally invasive surgical approach. This safety profile benefits from several procedure-specific instruments developed by our team. During intervertebral disc space preparation and cage implantation, the working cannula provides continuous and effective protection to neural structures. We recommend performing central canal decompression by resecting the ligamentum flavum after completing these key intradiscal steps, as this sequence can substantially reduce the risk of cerebrospinal fluid leakage or neurological injury. Furthermore, no surgical site infections were recorded in the Endo-PLIF group, whereas two cases of superficial surgical site infections were observed in the MIS-TLIF group, both of which achieved satisfactory healing after antibiotic therapy and wound management. A retrospective study by Bahir et al. (27) confirmed a lower surgical site infection rate in endoscopic lumbar fusion compared with MIS-TLIF, which is consistent with our findings. This finding may be attributed to the more minimally invasive nature of the endoscopic procedure and the continuous saline irrigation during surgery, which reduces wound contamination.

## 5 Conclusions

Both Endo-PLIF and MIS-TLIF achieve favorable clinical and radiographic outcomes in elderly patients with single-segment lumbar degenerative disease. Compared with MIS-TLIF, Endo-PLIF confers advantages including minimized damage to paraspinal muscles and bony structures, reduced intraoperative blood loss, more pronounced early postoperative pain relief, and facilitated postoperative recovery, thereby serving as a safer and more minimally invasive therapeutic option for elderly patients with lumbar degenerative diseases (LDD).

## 6 Limitations

This study is a single-center retrospective analysis with inherent limitations, including a limited sample size, potential selection bias, and a relatively short follow-up period. Moreover, the present study relies heavily on subjective clinical indicators and lacks objective evaluations, such as imaging-based assessments of paravertebral muscle quality and dynamic monitoring of perioperative biomarkers (e.g., creatine kinase). Additionally, the conventional threshold of 65 years was used to define elderly patients, a criterion that may warrant re-evaluation given the increasing global life expectancy. Future research should involve multi-center, large-sample prospective controlled trials with extended follow-up, incorporate more objective assessment parameters, and optimize the age definition for elderly populations. Such efforts would enable a more comprehensive and objective validation of the long-term efficacy and safety of the Endo-PLIF technique.

## Data Availability

The original contributions presented in the study are included in the article/Supplementary Material, further inquiries can be directed to the corresponding author.
